# ‘The false reporter will get a praise and the one who reported truth will be discouraged’: a qualitative study on intentional data falsification by frontline maternal and newborn healthcare workers in two regions in Ethiopia

**DOI:** 10.1136/bmjgh-2021-008260

**Published:** 2022-04-05

**Authors:** Abiy Seifu Estifanos, Rediet Gezahegn, Dorka Woldesenbet Keraga, Abiyou Kifle, Fanny Procureur, Zelee Hill

**Affiliations:** 1 Department of Reproductive, Family and Population Health, School of Public Health, Addis Ababa University, Addis Ababa, Ethiopia; 2 Institute for Healthcare Improvement, Addis Ababa, Ethiopia; 3 Institute for Global Health, University College London, London, UK

**Keywords:** Health systems, Maternal health, Qualitative study, Child health

## Abstract

**Introduction:**

Health Management Information Systems (HMIS) are vital to ensure accountability and for making decisions including for tracking the Sustainable Development Goals. The Ethiopia Health Sector Transformation Plan II includes preventing data falsification as a major strategic initiative and our study aimed to explore the reasons why healthcare providers intentionally falsify maternal and newborn health (MNH) data in two regions of Ethiopia.

**Methods:**

We conducted a qualitative study in two hospitals, four health centres and their associated health posts in Oromia and Amhara regions. We conducted 45 in-depth interviews with health facility managers, quality improvement (QI) focal persons, health information technicians, MNH care providers, Health Extension Workers and QI mentors. Data were collected in local languages and transcribed in English. During analysis we repeatedly read the transcripts, coded them inductively using NVivo V.12, and categorised the codes into themes.

**Results:**

Participants were hesitant to report personal data falsification but many reported that falsification is common and that they had experienced it in other facilities or had been told about it by other health workers. Falsification was mostly inflating the number of services provided (such as deliveries). Decreasing the number of deaths or reclassifying neonatal death into stillbirths was also reported. An overarching theme was that the health system focuses on, and rewards, the number of services provided over any other metric. This focus led to both system and individual level incentives for falsification and disincentives for accurate reporting.

**Conclusion:**

Our finding suggests that to reduce facility level data falsification policy makers might consider disentangling reward and punishments from the performance reports based on the routine HMIS data. Further studies examining the high-level drivers of falsification at regional, national and global levels and effective interventions to address the drivers of data falsification are needed.

**WHAT IS ALREADY KNOWN ON THIS TOPIC**

- Research on intentional data falsification of routine Health Management Information System (HMIS) data by frontline healthcare workers is limited, yet this is recognised as a major issue in some settings.

**WHAT THIS STUDY ADDS**

- Our study found that intentional data falsification by frontline health workers was reported as common.
- A system focus on service provision metrics underpins system and individual incentives for inaccurate, and disincentive for accurate, reporting.

**HOW THIS STUDY MIGHT AFFECT RESEARCH, PRACTICE AND/OR POLICY**

- Our data suggest that to improve the quality of HMIS data in Ethiopia more attention needs to be given to the system drivers of intentional data falsification.
- Further studies on high level drivers of falsification at national, regional and global levels along with their effective interventions are warranted.

## Introduction

A Health Management Information System (HMIS) is one of the WHO’s health system building blocks. A well-functioning HMIS ensures the production, analysis, dissemination and use of reliable and timely health information on health determinants, health systems performance and health status.^1^ This information is vital to facilitate evidence-based decision making at local, national and international levels^2^ and to track the performance on the Sustainable Development Goals.^3^

In Ethiopia, HMIS data are predominantly related to service delivery and are collected at all levels of the health systems. Data from health posts are sent in summary form to the health centre, where each department generates a summary of their indicators. Data are then sent from the health centre to the District Health Office who send it to zonal level, who send it on to the regional level.^4^ The Ministry of Health of Ethiopia has made substantial efforts to improve the quality and utility of routine HMIS data. The first Health Sector Transformation Plan (HSTP) had an ‘Information Revolution’ as one of its four transformation agendas. This transformation included adopting the web-based District Health Information Software 2, bolstering the data verification and feedback system, creating a data use culture and enhancing data visibility and access.^5 6^

Despite these efforts the second HSTP identified poor data quality as a system weakness^7^; this was also reported by several studies which characterised Ethiopia’s HMIS as providing incomplete, inaccurate and untimely data with low utilisation for decision making.^8–13^ Possible reasons for the poor quality data include poor support by the facility management, poor supervision and feedback, high workload, staff turnover, lack of tools, low competency, low motivation for accurate reporting, carelessness, lack of accountability for false reports, manipulating data for competition, and a lack of a separate and responsible unit for routine HMIS.^10 13^

While the causes of poor quality HMIS data are multi-factorial^14^ the Ethiopian Government has identified the need to prevent data falsification as a major strategic initiative within HSTP II.^15^ Intentional data falsification is rarely mentioned or studied but may be relatively common driven by pressure from leaders, a fear of a facility appearing under-used, links between performance/patient numbers and funding or the provision of equipment and medicines, and a lack of accountability.^13 16–18^ Given the potential role of data falsification in data quality, the limited data on the topic and the focus on falsification in HSTP II our study aims to explore the reasons why healthcare providers intentionally falsify MNH data in two regions of Ethiopia.

## Methods

### Study settings

We conducted a qualitative study between July and August 2018 in Amhara and Oromia regions of Ethiopia. Oromia and Amhara regions are the most populous in Ethiopia with an estimated 2020 population of 38 and 22 million, respectively. Together the two regions constitute around 60% of the total population of Ethiopia.^19^ They contribute the largest number of maternal and neonatal deaths of Ethiopia’s 11 regions and rank 3rd and 4th in terms of their neonatal mortality rate. Their neonatal mortality rate is 46 and 39 deaths per 1000 live births for Oromia and Amhara, respectively,^20^ and their estimated maternal mortality ratio 520 and 369 deaths per 100 000 live births.^21^

This study was part of a larger qualitative study to explore the functioning of the prototype phase of an MNH quality improvement (QI) initiatives being implemented by the Ministry of Health supported by the Institute of Healthcare Improvement.^22^ The QI intervention included the formation of learning collaboratives at woreda (district) level, the formation of facility MNH QI teams to plan, implement and monitor QI projects and change ideas and collaborative level learning sessions to share experiences, build clinical skill and develop and review change ideas. QI teams were supported through visits by QI mentors who, among providing other support, validated HMIS data and worked with the team to improve accuracy through trust building, training and feedback.^23^

### Sampling and study participants

From each region we selected one woreda, within which we selected the hospital, a less accessible health centre located at a remote village with access by a rough road and a more accessible health centre located near a main road. Both health centres were ‘typical’ in that they had no unusual additional interventions or staff in place and were in a typical geographic area for the woreda. The two hospitals were better equipped and staffed than the health centres, and two of the health centres had intermittent electricity and one had no running water. One was an upgraded health post and had poor building quality and lacked equipment.

Within each facility we conducted in-depth interviews (IDI) with 5–8 participants including the health centre/department manager, the MCH focal person, the health information technicians (HITs), MNH clinical care providers (midwives, nurses and health officer) and HEWs. To be eligible for interviews participants needed to have some experience of the QI project either through attending learning sessions or being part of the facility QI team. We also interviewed three QI mentors. In the majority of cases, participants were identified by the data collectors as facilities were small. When needed, the facility head and the QI mentors helped identify the MCH focal person, the HIT and other who were involved in QI, but they were not directly involved in approaching or recruiting participants. Identified participants were approached at the facility by one of the qualitative interviewers who checked that they were not busy with clinical work and found a private place to take consent and conduct the interview. IDIs were conducted in Amharic or Afaan Oromo languages, and digitally recorded. In total, we interviewed 45 participants. The majority of the participants were females who had worked in the health facilities for 1–4 years. Table 1 summarises the study participants.

**Table 1:** Background characteristics of the study participants (n=45)

Characteristics	Frequency
Sex*	
Female	26
Male	16
Facility type†	
Hospital	11
Health centre	31
Job title	
Manager of health centre/department	7
Maternal and child health focal person	4
Midwife/nurse	11
Health officer	3
Health information technician	4
Laboratory technician	1
Health extension worker	12
QI mentor	3
Time at the facility‡	
<1 year	3
1–4 years	28
5–9 years	10
≥10 years	2

*Three missed data.

†Excludes woreda/Institute of Healthcare Improvement mentors.

‡Two missed data.

QI, quality improvement.

### Data collection: IDI

Six trained and experienced qualitative interviewers conducted the IDIs using pretested semi-structured interview guides. The guides collected data on the QI intervention but also contained questions related to data quality with participants asked explicitly about falsification: ‘There can be many reasons why health facilities exaggerate their performance. Can you share any experiences of this?’. During the interviews the data collectors took field notes. Daily debriefing meetings with study investigators were organised to discuss the adequacy of the topic guides, review and give feedback on transcripts, increase reflexivity and assess saturation.

### Data analysis

Analysis began during the daily de-briefs where the importance of data falsification first emerged and was discussed within data collection team. Using the grounded theory approach,^24^ the research team developed hypotheses as we reviewed all transcripts and extracted all data related to data falsification. Indeed, extracts centred on particular incidents or behaviours were coded inductively through in depth and repeated reading to identify a first set of themes and codes followed by a thorough coding using NVivo software V.12. The research team met regularly during the analysis to discuss the emerging codes, whether any codes should be merged and to discuss patterns, links and contradictions in the data.

### Patient and public involvement

This study was conducted to assess the perspectives of health workers involved in the planning and implementation maternal and newborn healthcare services and did not involve patients or the public in the design, conduct, reporting, or dissemination of the study findings.

## Results

In this section, we first present descriptive data on what falsification participants reported occurred. We then present five themes related to reasons data are falsified: ‘system’s focus on numbers’, ‘system level incentives to falsify data’, ‘system disincentives to report actual data’, ‘individual incentives to falsify data’ and ‘individual disincentives to report actual data’.

### Data falsification

Most participants reported that there was no data falsification in their current facility. This was reported as a recent change and was often linked to the QI intervention as this included data verification, supervision from the mentors, being taught the importance of accurate data and using the data for decision making.

It [data falsification] has changed since the QI initiative …. Health professionals are encouraged to send the actual report [representing] the actual performance …. they started to report what they actually [have] done. I think we have seen a change after the QI initiative started. (ID07, nurse)

We noted some hesitancy talking about personal data falsification with many participants stating they are not liars or that falsification is not in their nature. One participant who reported that they had personally falsified data requested the tape recording be switched off highlighting that they viewed this as a sensitive topic. Despite reporting that it did not happen in their facility many participants reported that falsification was common and that they had seen it in other facilities or had been told about it by other workers:

… I never did like this [falsified data] in [my] history. I am not a liar in nature. If there is death, I always record the death …. However, my friends who work in other facilities tell me what they do [falsify data] when they are told to increase the number of deliveries …. (ID02, midwife)

The most commonly reported falsification was inflating the number of healthcare services provided, particularly deliveries, but there were also reports of decreasing the number of deaths or reclassifying neonatal deaths as stillbirths.

We don’t do such a thing [reclassifying neonatal death into stillbirth] … But sometimes it may occur. For example, a midwife may say it [the neonatal death] is stillbirth or IUFD [intra uterine fetal death] not to be reprimand or criminalized for a child who died because of poor follow up. Such practices are avoided after QI came to this hospital. (ID14, midwife)

Falsification was reported to be done by heath workers and by facility managers when they generate and submitted reports.

It was with guess that it [reporting form] was filled before. It was only the [manager] who filled this form. I [the manager] was filling this form alone. False reports were present … (ID06, health facility manager)

### Reasons for data falsification

**Figure 1:**
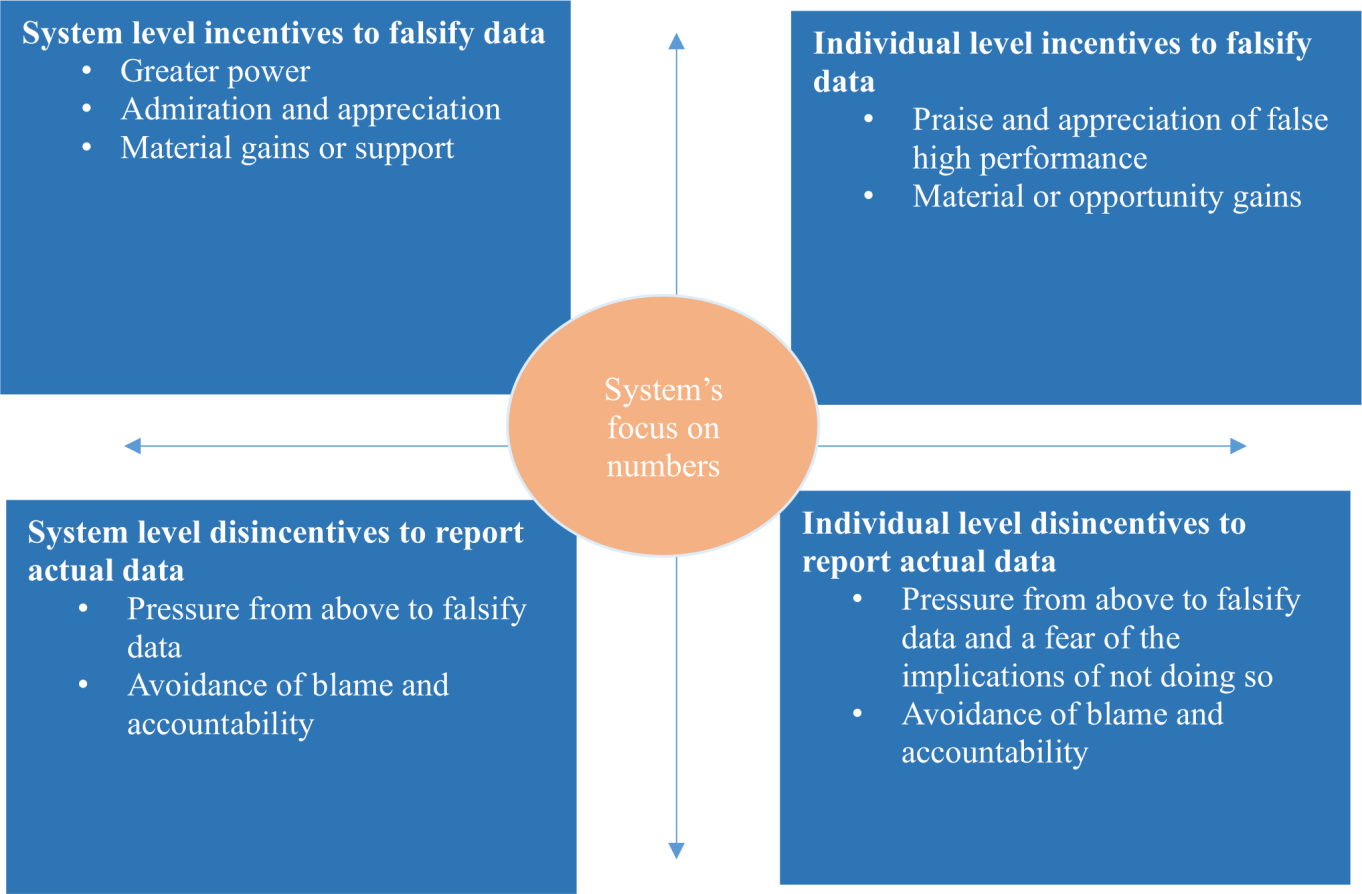
Reasons for intentional data falsification by frontline maternal and newborn health care workers, Ethiopia.

Although most participants did not report a practice of data falsification in their facilities they reported several reasons for data falsification in other facilities (figure 1). An overarching theme was that the health system focuses on, and rewards, the numbers of services provided over any other metric. This focus led to both system and individual level incentives for falsification and disincentives for accurate reporting. Incentives include praise and recognition of the facility or the individual, benefits for the facility such as increased power or material support and for the individual such as bribes or better educational opportunities. Disincentives included explicit pressure to falsify from those above you, this was from woreda officials at the system level and facility managers at the individual level, with a fear of a negative impact for those that did not comply. There was also a culture of blame for poor performance at facility and individual level, and a fear among individuals that they would be held accountable for poor clinical outcomes.

### System’s focus on numbers

A key theme was that there was a culture of falsification throughout the health system driven by the need to show high performance, which was judged by numbers, and was perceived to be desired by those above. Connected with this was a culture of blame for those who performed poorly.

Facilities exaggerate data because of the system, if the bosses are looking for high performance, the health professionals report high numbers to get appreciation from their bosses. Those who have reported false reports are appreciated and those who report lower numbers are blamed. So, this system encouraged the facilities [to] overreport their performance (ID07, nurse)

Even if I assist the delivery of a single woman they ask me to report it as ten [women] …. Once, I reported eight deliveries per month and the manager from health center and the woreda shouted at me and they say [to] me ‘you have some political problem’ (ID31, midwife)

### System level incentives to falsify data

The number driven system led facilities and officials to exaggerate their performance so they would rank highly to create a positive image for their facilities compared with other facilities and to gain power or recognition, admiration, appreciation and praise for the facility.

Previously, to compete with other health center …. maybe to not lose their position; they tried to exaggerate the data. Due to these reasons they reported false reports (ID05, Health officer)

Sometimes they need power. Sometimes not to be insulted. And sometimes for example if we need to increase the name [improve the image] of our woreda, we overstate our data. This is to get our health center recognized. (ID11, HEW)

Exaggerated numbers could also lead to material gains such as receiving more ‘support’ or being eligible for a facility upgrade. For example, study participants from the hospitals identified the need to demonstrate high performance in relation to the number of services provided to upgrade the level of their hospital.

…; it is when there are a lot of cases or[we] provide services for many clients that the hospital gets support …. For example, it was said this hospital should upgrade to a referral hospital …. It was asked to present a report on the number of cases …. It is when they want to get support from the higher health bureau that they may exaggerate the numbers…. (ID12, midwife)

### System level disincentives to report actual data

Study participants reported they had experienced, seen or heard about pressure being exerted on health workers to falsify data using terms such as ‘forced’, ‘pushed’ or ‘made’. Those exerting pressure were often described as ‘they’, but several participants clarified that they were referring to facility, woreda or zonal managers.

…. They push us to record what has not been done. For example, if there are 11 deliveries, they push us to record it as 21 deliveries. They push us to lie … (ID10, midwife)

Participants described a culture of blame where health workers and managers would be held accountable for ‘poor’ performance even when this was caused by factors outside of their control.

…. when it is said facility delivery has increased in other woredas, they say you are not performing well, but we are doing what we are supposed to do.…. (ID10, midwife)

### Individual level incentives to falsify data

Incentives to falsify information at the individual level included appreciation and praise for false high performance, tangible benefits through bribes and greater transfer and educational opportunities.

Participants reported that health officials often praise/appreciate false high performance and sometimes pay money to ensure exaggerate performance.

… Let’s say two people were given ten works [tasks] each. But, in truth both of them did two out of ten, and one reported eight to be above 50% whereas the other reported exactly two; then your manager and people from the woreda blame you like you are not working, you are inefficient and such like, words that discourage you, despite your hard work to get even that two out of ten. So, the false reporter will get praise and the one who reported the truth will be discouraged …. (ID15, HEW)

… I know one health professional who quarreled with the [manager] of the woreda health office due to this [falsifying data]. He argued with him when he ordered him to increase the report. The other midwife received money and did what the [manager] said … (ID02, midwife)

Participants reported that healthcare workers who got education opportunity or transfer to a better health facility were those who manipulated the data to report high healthcare service provision performance.

… there was a summer education opportunity that was given to the health professional providing the most delivery services. Due to this, there was false recording of facility deliveries, even for mothers who had had home delivery …. (ID16, MCH focal person)

… If you do a good job, you get the chance of a transfer from one area to another area. They were thinking about these kinds of issues. That is why they reported the false report. (ID18, midwife)

### Individual level disincentives to report actual data

At individual level there are different disincentives to report actual performance data including a desire not to be directly held accountable or blamed for poor performance, and not wanting to make their life difficult or fear of losing their job.

Participants stated that healthcare providers do not want to be blamed for low performance in provision of healthcare services and/or for reporting the actual high number of poor health outcomes. They also have a fear of being held directly accountable for poor clinical outcomes causing non-reporting or to misclassification of deaths.

…. It was eleven neonatal deaths [that were] reported in six months in this cluster, but what was reported was only six deaths. They have deducted five deaths because they want to exaggerate their performance. They do not want to be blamed. They hide information so as not to be blamed and insulted. It is the reason for false reporting; if there are a high number of deaths, the midwives will be blamed or insulted…. (ID04, HEW)

Some respondents mentioned fear of losing one’s job or stable income as a reason to falsify the MNH care service data.

… they are afraid of losing their job, so that, they tend to lie. The first reason for falsifying data is, to eat the available Enjera [Ethiopian flat bread] peacefully…. (ID19, QI mentor)

Many participants reported that most health workers do not want to lie, and have a responsibility not to do so, and sometimes argued with their peers and superiors about falsification, but did so in the context of a system that encouraged and rewarded falsification.

## Discussion

Our study employed qualitative methods to explore reasons why healthcare providers and managers intentionally falsify routine HMIS MNH data. Few participants reported data falsification in their facilities. This may be due to social desirability bias, changes over time or the impact of the QI intervention. Most respondents reported that intentional MNH data falsification is a problem in other facilities and we found this to be driven by the system’s focus on numbers which resulted in the system and individual level incentives and disincentives.

The data generated through the routine HMIS is widely criticised for its poor quality limiting its use for planning and monitoring the performance of health programmes.^10 14 16^ Frameworks on HMIS data quality and use focus on addressing the health system, organisational, and individual factors that affect the generation and use of quality routine HMIS data. Despite its potential importance data falsification is not included in the data QI frameworks and data QI initiatives.^14^ The Ethiopian HSTP II recognises the importance of data falsification by including a national movement to prevent data falsification as one of its major initiatives,^7^ but we found little written about the drivers of falsification in Ethiopia, or more widely. Our study brought these drivers to light which may facilitate the development of strategies to address the problem as well as leading to conducting further studies looking at the high-level drivers of routine HMIS data falsification at national, regional and global levels.

Our data suggest the system’s focus on numbers underpins the system and individual reasons for data falsification. This focus could be politically driven by the country’s desire to receive funding from donors.^16^ In addition, at the time this study was conducted, Ethiopia had a government that silenced discussion on politically sensitive issues leading to increased social desirability bias including in the health system performance reporting.^25^ Although cultural transformation in data falsification at the lower level of the health system is unlikely in the short term, the acknowledgement of the problem in HSTP II reflects an important step that the current government of Ethiopia is taking towards addressing this systemic problem.

Although not the focus of this study our data, and those from other studies,^23^ suggest that QI interventions may reduce data falsification by motivating health workers to report accurate data through data verification, supervision, being taught the importance of accurate data and using the data for decision making. However, QI interventions may not address the system and individual level drivers identified in this study. To address these drivers their needs to be a disentangling of the organisational and individual reward and punishment measures from the HMIS based performance reports.^16^ In addition, further research on effective interventions to address the system and individual level factors driving the data falsification are needed.

Although we included different groups of respondents who had different experiences of collecting, summarising and submitting data our study was conducted within facilities participating in a QI intervention which may limit its transferability. However, most of the reports of falsifications were about participants’ experiences or knowledge of other facilities not participating in the QI intervention. We did not interview health officials at the woreda, zonal, regional or national level which would have provided a broader perspective on, and a deeper understanding of, data falsification at the systems level. Although we trained our data collectors on rapport building and on strategies to reduce social desirability this is likely to have persisted due to the sensitive nature of the topic.

## Conclusion

Our study contributes to the global discussion on quality of data in the routine HMIS by offering evidence on the drivers of intentional data falsification by frontline maternal and newborn health providers in resource limited settings. Underpinned by a system’s focus on numbers, reasons for data falsification include system and individual level incentives and disincentives. To improve the validity of routine HMIS data we recommend data QI frameworks and interventions reflect on the reasons for falsification identified through our study.

## Data Availability

Data are available upon reasonable request. The data are confidential considering that data falsification is a sensitive topic and participants could be identified if their interviews are read in full. A formal request needs to be made and a data sharing agreement will have to be made before sharing the data.
